# Higher low back and neck pain in final year Swiss health professions’ students: worrying susceptibilities identified in a multi-centre comparison to the national population

**DOI:** 10.1186/s12889-018-6105-2

**Published:** 2018-10-19

**Authors:** Rebecca J. Crawford, Thomas Volken, René Schaffert, Thomas Bucher

**Affiliations:** 1 Faculty of Health Professions, Curtin University, Perth, Australia; 2 Institute for Health Sciences, Zurich University of Applied Sciences, Winterthur, Switzerland; 3 Specialist Unit for Quality Management and Evaluation, Zurich University of Applied Sciences, Winterthur, Switzerland

**Keywords:** Low back pain, Neck pain, Self-reported health, Health profession, Students, Switzerland, Swiss health survey

## Abstract

**Background:**

Low back pain (LBP) and neck pain (NP) are of considerable socioeconomic burden. Considering the escalating demand on health services that LBP and NP have globally, they represent an arguably unsustainable drain on resources with the projected increased demand secondary to an ageing population. Identifying populations at risk for LBP and NP may inform public health prevention strategies. Health professions’ (HP) students may be more susceptible due to their demographic factors and potentially risky postural demands of their education and formative clinical practice. The aim of our study was to compare self-reported LBP and NP of HP students with the general and stratified Swiss population to identify their prevalence. In addition, we compared the prevalence of LBP and NP in students studying different professions in order to identify whether susceptibilities exist.

**Methods:**

In this cross-sectional study, self-reported LBP and NP reported by final-year HP students (*n* = 1848) were compared with the Swiss national population aged ≥15 years living in private households (*n* = 21,597). Binary regression models estimated crude prevalence and prevalence adjusted for age, gender, and education. Design-based F-Tests assessed differences between students and the Swiss population.

**Results:**

Crude, overall four-week (4w) prevalence (mean (95% CIs)) for LBP was 61.0% (58.4–63.5) in all HP students versus 40.0% (39.2–40.9) in the Swiss population. Female HP students aged 21–30 years (63.3% (60.5–66.1)) reported higher LBP than the same-aged Swiss female population with secondary (43.7% (39.5–47.9)) or tertiary (36.6% (30.8–42.9)) education. Crude, overall 4w prevalence for NP was 59.8% (57.2–62.3) in all HP students versus 36.4% (35.6–37.3) in the Swiss population. Female health professions’ students aged 21–30 years reported higher NP (63.2% (60.4–66.0)) than the same-aged Swiss female population with secondary (36.6% (32.7–40.8)) or tertiary (35.4% (29.6–41.8)) education. The inter-professional differences shown indicate midwifery to be most susceptible to reporting both conditions.

**Conclusions:**

Considerably higher LBP and NP were reported by final year HP students when compared with the general and stratified Swiss population. Worrying inter-professional susceptibilities were shown and reveal the need for further explanatory studies. Measures to reduce complex health problems like LBP and NP should be introduced into curricula in order to optimize the longevity of clinical careers and to protect the future HP workforce.

## Background

Low back pain (LBP) and neck pain (NP) are among the most prevalent and disabling diseases globally [1] impacting individuals at a personal level, and more widely, the public at large [2, 3]. Like the rest of the world, LBP is Switzerland’s most disabling disease; NP ranks third, which is one rank above its global position [1, 4] and may suggest relatively more NP in the Swiss population [5]. These common musculoskeletal conditions have shown the greatest growth in health spending in the last two decades [3, 6] with an even higher economic burden projected [7] to coincide with the world’s ageing population [2–4, 8, 9]. The variety and uptake of treatments for LBP in particular have increased [6, 10]; yet, without appreciable reduction in the problem [1, 3, 9]. Novel, effective, and resource-efficient strategies are urgently needed to mitigate the economic, social, and personal impact of LBP and NP [3, 11, 12]. Identifying and understanding causation for populations at risk for developing these conditions is an important step toward promoting and instigating preventative and mitigating public health measures.

Both LBP and NP are common, with point and yearly prevalence (LBP/NP) around 18/14% and 38/26%, respectively. However, incidence and prevalence vary considerably with the population studied and therefore mean estimates should be considered against equitable comparators [13, 14]. A systematic review showed the incidence of LBP to be highest for those aged in their twenties, and an overall increasing prevalence toward the 60–65 year age-group, with a gradual decline thereafter [13]. Worryingly in terms of sustained burden, most people who experience activity-limiting LBP have recurrence within a year [13] and are vulnerable to progression to chronicity as a considerably tenacious problem [15]. Other common risk factors for LBP include low educational status, mental health problems, job dissatisfaction, and poor workplace support [13]. For NP, there is an increased risk approaching the age-group 35–49 years, with a gradual decline thereafter [14]. The prevalence of NP is generally higher in women than men, in high-income countries, and particularly more prevalent in office-workers [4, 14]. In Switzerland, the prevalence and cost-burden of LBP and NP appear on the high-end of global estimates [5, 16], and are likely influenced by several environmental and personal factors.

Considering these known risk factors, health profession (HP) students may be vulnerable to LBP and NP as they are a predominantly young, female demographic, [17, 18] and who likely spend considerable time in desk-based study postures. Educational programmes for HPs in Switzerland consist of about 1800 study hours per year involving 40% classroom teaching and 60% self-directed study [19]. Literature suggests that the prevalence of LBP and NP in students and practicing HPs appreciably exceeds mean estimates [20–27]. Furthermore, findings indicate that evidence for inter-professional susceptibilities exist [20–22]. Commencing professional careers with existing LBP or NP may threaten longevity and productivity at work, which is worrying in light of the World Health Organization identifying a mounting global HP work-force shortage [28]. This concern has also been confirmed for Switzerland where almost 20% more HPs are needed by 2025 to match projected demand [29]. In order to face the work-force challenge this presents, it will be necessary to recruit more students and also to reduce attrition from university and clinical practice that LBP and NP may contribute to.

HP students were surveyed in an overarching study to gain insights regarding their entry into the profession and to identify student competencies and factors that optimize retention in the work force. The survey comprised questions on health status, and specific questions on LBP and NP were then employed in the current study. Our first aim was to examine the prevalence of LBP and NP in HP students in comparison to the Swiss population. Our second aim was to determine whether inter-professional differences in prevalence existed, such that susceptibilities within the Swiss HP student population might be better understood and to enable targeted management. As far as we are aware, our study is the first to compare HP students’ self-reported LBP and NP to the national population, and specifically to stratified peers. We deemed this national focus as essential in effectively understanding the gravity of the problem at a local level in order to provide evidence for public health and institutional changes.

## Methods

### Study design

The study is a comparative, secondary analysis of a population-based cross-sectional health survey (2012) and final year health professions’ student surveys (2016 and 2017) undertaken in Switzerland.

### Study population and data

The Swiss Health Survey (SHS) undertaken by the Swiss Federal Statistical Office (SFSO) is a nationwide survey on health status, health service utilization, and health-related behavior. The SHS employs telephone interviews and subsequent written questionnaires; it was first conducted in 1992 and is repeated every five years. For each survey year, a multistage probability sample is drawn of the permanent resident (including foreign nationals) population in Switzerland after stratification by the three predominant language/geographic regions (German, French, and Italian). Samples include individuals’ aged 15 years or older living in private households and excluding those living in institutions, i.e. hospitals, homes for the elderly, prisons, monasteries, and military barracks; only subjects conversant in either of the three languages are surveyed. Data were collected and administered by the SFSO under the regulation of the Federal Statistics Act (FSA) of 1992, which is a framework of law dedicated to federal data collection, data protection, and data security. Participants provide informed consent, which accommodates all future use of the data for research (FSA, 1992).

For our study, we obtained the most recent SHS data (2012). The respective net sample size comprised *n* = 21,597 respondents, representing 6,838,268 subjects in the general population. Data for HP students were derived from the National Survey of Final Year HP Students (National Graduate Survey of Health Professionals from Universities of Applied Sciences; Nat-ABBE). The Nat-ABBE is a nationwide census survey of final year HP students at six universities within the three major language regions (German, French, and Italian). The Nat-ABBE is part of a nationwide collaboration of Universities of Applied Sciences (https://www.cnhw.ch/en/) to develop a competence network to counter projected shortages in the health workforce. While the main focus of the Nat-ABBE encompasses education and professional development, it also comprises several questions on health status, and health-related behavior. The Nat-ABBE employs written online questionnaires; it was first conducted in 2016 and is repeated every year. We obtained Nat-ABBE data for the years 2016 and 2017 with a respective sample size of *n* = 1980. This sample was reduced to include only full-time students and students of large faculties, i.e. midwifery, nursing, nutritional sciences, occupational therapy, and physiotherapy (*n* = 1848). Excluded subjects (*n* = 132) comprised all students from Medical Radiology (*n* = 47) because this subject can only be studied in the French speaking part of Switzerland. Moreover, students of nursing and midwifery with a nursing diploma were also excluded (*n* = 85) because they worked already in the healthcare system, they studied part-time, and were much older than their fellow students.

For the comparison between the general Swiss population and HP students, data were pooled yielding an initial combined sample size of *n* = 23,445. Moreover, we extracted three demographically-stratified samples of female participants aged 21–30 years with secondary (*n* = 848), tertiary (*n* = 386), and secondary or tertiary education (*n* = 1234) from the SHS to match the corresponding female HP students (*n* = 1501) yielding a restricted pooled sample of *n* = 2349; *n* = 1887; and *n* = 2735. The comparison of HP students who were about to complete tertiary education with SHS respondents in the three respective samples served to assess potential education related differences in pain prevalence. More specifically, SHS respondents indicated their education level but we lacked information on whether they were completing a higher level of education at that time, i.e. respondents who completed secondary education may or may not have been studying at the tertiary level at the time the SHS was administered. Consequently, we used the three restricted samples to assess whether HP students’ pain prevalence were more similar to SHS respondents who completed tertiary education or to SHS respondents who completed secondary education or were more similar to a mixture of SHS respondents with either completed secondary or tertiary education.

### Outcomes: Prevalence of LBP and NP

Self-reported LBP and NP, the target outcomes of this study, were derived from self-reported data. SHS participants were confronted with a list of health problems, including LBP and NP, and were asked to report for each health problem whether they had experienced it (Question: “I will read out different health problems. Please tell me for each of these health problems whether in the past 4 weeks you have had it”). Responses were captured using a three-point ordinal scale (no, a little bit, strong). Similarly, the Nat-ABBE asked respondents to report health problems (Question: “In the past year, have you had one or more of the following health problems?”); responses were captured using a four-point ordinal scale (no, rarely, occasionally, often). Unfortunately, the SHS wording of the question (“Bitte sagen Sie mir jedes Mal, ob Sie das in den letzten 4 Wochen überhaupt nicht, ein bisschen oder stark gehabt haben.”) is not very precise and its closeness to colloquial language makes it difficult to judge whether it referrers to the frequency or intensity of pain. Similarly, the three categories do not allow for a final judgment of what respondents had in mind when they answered the SHS question because the categories “a little bit” and “strong” may also refer to either frequency or intensity in many Swiss dialects. In contrast, the Nat-ABBE clearly refers to pain frequency. Despite this ambiguity and the difference in the number of response categories, we feel it is safe to assume that the common category “no” describes absence of pain in general, i.e. frequency and intensity of pain is zero while any other category describes the presence of pain in general. Therefore, we derived a subject-specific binary outcome for LBP and NP (yes/no), indicating the presence or absence of pain. Prevalence of LBP and NP was conceived as the proportion of respondents reporting pain. While the dichotomization of response categories for LBP and NP in our study was primarily driven by the need to make outcomes more comparable across the two surveys, it is also not unusual for prevalence studies of LBP and NP to use dichotomous outcomes [30]. The systematic review of Hoy et al. [31] show that the majority of studies (661 of 893) do not specify the minimum episode duration necessary for inclusion, while one day was the most used when reported. As such, prevalence of LBP and NP used in our study is comparable to other studies. However, our self-reported prevalence of LBP and NP may differ from prevalence estimates in which minimum episode duration was specified [31].

Collapsing of categorical variables, whilst valid, involves loss of information and may lead to reduction in efficiency in the statistical analysis under consideration [32]. Moreover, changing outcome categories can affect the effect estimates as well as the inferences drawn from the data [33, 34]. In order to address the latter issue and justify the dichotomization of our dependent variables, we assessed the association between age, gender, education, and LBP and NP respectively [35]. We compared the results obtained for the dichotomous variables using a logistic model with the results obtained for the original ordered categorical variables using a cumulative odds model (results not shown). The results yielded by the logistic model were confirmed by the alternative cumulative odds model, which incorporated the original three (SHS) and four (Nat-ABBE) ordered categories of LBP and NP, i.e. we found similarity of results regarding the size and statistical significance of effects. Furthermore, collapsing the original dependent variables into two instead of three categories was motivated by the need to derive clearly distinguishable categories from two different scales and simultaneously provide measures of LBP and NP, which achieve the best possible comparability with other studies, i.e. roughly 74% of the studies considered by Hoy et al. [31] reported prevalence of LBP based on dichotomous outcome measures (presence/absence of LBP).

Prevalence differs substantially according to prevalence period, i.e. point, four-week, yearly or lifetime. In students, yearly prevalence of LBP and NP was assessed (via the Nat-ABBE), while the national population was assessed (via the SHS) based on four-week prevalence. We derived frequency weights based on a well-cited systematic review of LBP, reporting and comparing 145 four-week and 271 yearly prevalence of LBP [31], to estimate comparable four-week prevalence in students. The systematic review found that on average, yearly prevalence was 1.25 times higher than four-week prevalence. Frequency weights were calculated as following:

1 $$ {\omega}_1={1.25}^{-1} $$

2 $$ {\omega}_2=\frac{N-\left({\omega}_1\bullet {n}_1\right)}{n_2} $$

3 $$ N=\sum {\omega}_i $$

Where:

N is to total number of students in the sample

n_1_ is the number of students with the pain condition

n_2_ is the number of students without the pain condition

ω_1_ is the frequency weight for students with the pain condition

ω_2_ is the frequency weight for students without the pain condition

### Frequentist and Bayesian statistical approaches

Binary regression models were employed to estimate crude and adjusted prevalence. Adjustment comprised age, gender, and education. We used a conventional frequentist statistical approach for the comparison between HP students and the general Swiss population. However, a Bayesian statistical approach was employed for the comparison among HP students because of its flexibility to derive many different models, i.e. dyadic comparisons among the five health professions, from the posterior distribution. The posterior distribution was determined using Markov-Chain-Monte-Carlo (MCMC) sampling. In order to assess convergence, we initially used 4 chains with 4000 iterations and monitored the corresponding trace plots. For the final estimates, a single chain with 20,000 iterations was used. The first 2000 iterations were discarded (burn-in phase). Non-informative priors, i.e. N(0, 5), were used for all parameters in the binary models.

### Statistical analysis

We used R Version 3.4.3 (R Foundation for Statistical Computing, Vienna, Austria), the package *‘survey’* [36, 37], and Stan [38] for statistical analyses. We reported estimated LBP and NP prevalence with corresponding 95% confidence intervals (95% CI) or 95% highest posterior density intervals (95% HPDI), respectively. Differences between the national population and students were assessed using design-based F-tests, which take into account the complex survey structure of the SHS [39]. Statistical significance was established at *p* < 0.05 [40]. Differences among students were assessed using predicted posterior mean differences in pain prevalence between student groups with corresponding 95% HPDI.

## Results

### Socio-demographic characteristics of respondents

Sociodemographic and health status characteristics of the initial combined sample are presented in Table 1. Not surprisingly, HP students differed substantially and significantly from the general population with respect to sociodemographics. HP students were younger (age 25.0 ± 3.9) than the general population (age 47.4 ± 18.8), more likely female (88.1% vs 51.0%), and represented a homogenous group with respect to their highest level of education (100% secondary education).

**Table 1:** Socio-demographic characteristics and prevalence of back pain and neck pain in the Swiss Health Survey^a^ and in the survey of final year health professions students

Variable	SHS	Nat-ABBE	P
Back pain (%)			0.000
No	60.0	39.0	
Yes	40.0	61.0	
Neck pain (%)			0.000
No	63.6	40.2	
Yes	36.4	59.8	
Gender (%)			0.000
Women	51.0	88.1	
Men	49.0	11.9	
Age group (%)			0.000
< 21	7.2	0.1	
21–30	15.1	94.7	
31–40	15.8	3.5	
41–50	20.3	1.5	
> 50	41.6	0.3	
Education (%)			0.000
Primary	18.0	0.0	
Secondary	53.4	100.0	
Tertiary	28.6	0.0	
Sample size	21,597	1848	
Population size	6,838,268		

^a^Percentages based on population weighted data. Four week prevalence for back pain and neck pain. *P*-values from design-based F-test

Source: Swiss Federal Statistical Office, Swiss Health Survey (SHS) 2012. Nat-ABBE coordination group, National Graduate Survey of Health Professionals from Universities of Applied Sciences 2016, 2017 (Nat-ABBE)

### Four-week prevalence in health professions’ students and in the general population

The crude overall four-week prevalence (mean (95% CIs)) for LBP in all HP students was 61.0% (58.4–63.5) versus 40.0% (39.2–40.9) in the general Swiss population (Table 1). Similarly, the crude overall prevalence for NP was significantly higher in all HP students (59.8% (57.2–62.3)) than in the general Swiss population (36.4% (35.6–37.3)). In order to adjust for age, gender, and education, we restricted the HP student sample to women aged 21–30 years (all with completed secondary education) and compared it with three corresponding restricted SHS samples of women in the same age group. The first SHS sample comprised only woman with completed secondary education, the second comprised only woman with completed tertiary education and the third included women who had either completed secondary or tertiary education. These three samples were used to comprehensively reflect SHS cases most likely to match the students. HP students had substantially higher prevalence of LBP and NP as compared to all three restricted SHS samples. Female HP students aged 21–30 years 63.3% (60.5–66.1)) reported higher LBP than the same-aged Swiss female population with secondary (43.7% (39.5–47.9)), tertiary (36.6% (30.8–42.9)), and secondary or tertiary (41.4% (38.0–45.0)) education. Moreover, female HP students aged 21–30 years reported higher NP (63.2% (60.4–66.0)) than the same-aged Swiss female population with secondary (36.6% (32.7–40.8)), tertiary (35.4% (29.6–41.8)), and secondary or tertiary (36.2% (32.9–39.7)) education.

### Yearly prevalence and differences of yearly prevalence among HP students

**Fig. 1:**
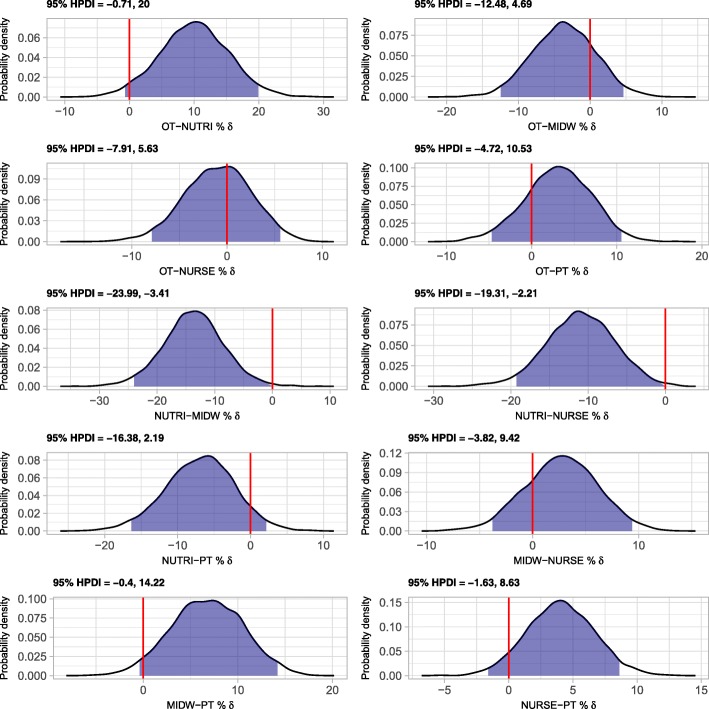
Probability density of differences in adjusted yearly prevalences of low back pain among health professions’ students. 95% HPDI: 95% highest posterior density interval. Source: Nat-ABBE coordination group, National Graduate Survey of Health Professionals from Universities of Applied Sciences 2016, 2017

The adjusted yearly prevalence of LBP (mean (95% HPDI)) was 76.4% (74.4–78.3) in the total sample of HP students (Table 2). We found the highest adjusted yearly prevalence of LBP in students of midwifery (80.6% (74.4–86.4)), followed by students of nursing (77.9% (75.7–80.1)), and students of occupational therapy (77.0% (71.6–82)). Students of nutritional sciences had the lowest prevalence (67.0% (58.7–75.1)). Differences in prevalence were substantial between students of nutritional sciences as compared to students of occupational therapy, midwifery, and nursing (Fig. 1) for which the probability of observing no difference or a negative difference, i.e. a lower prevalence as compared to students of nutritional sciences, was only 0.031, 0.005, and 0.005, respectively. Likewise, given the data and the model, the probability of observing a lower adjusted yearly prevalence of LBP in students of midwifery as compared to students of physiotherapy was 0.039.

**Table 2:** Yearly prevalence of low back pain in final year health professions students by profession

Profession	Crude prevalence (%)	95% HPDI	Adjusted prevalence (%)	95% HPDI
Occupational Therapy	77.1	70.9–83.3	77.0	71.6–82.0
Nutritional Sciences	65.9	58.3–73.6	67.0	58.7–75.1
Midwifery	81.1	75.0–86.2	80.6	74.4–86.4
Nursing	77.0	74.3–79.6	77.9	75.7–80.1
Physiotherapy	72.6	68.5–76.7	73.9	69.7–78.2
All	75.6	73.6–77.6	76.4	74.4–78.3

Adjusted prevalences: adjusted for age and gender. 95% HPDI: 95% highest posterior density interval

Source: Nat-ABBE coordination group, National Graduate Survey of Health Professionals from Universities of Applied Sciences 2016, 2017

**Fig. 2:**
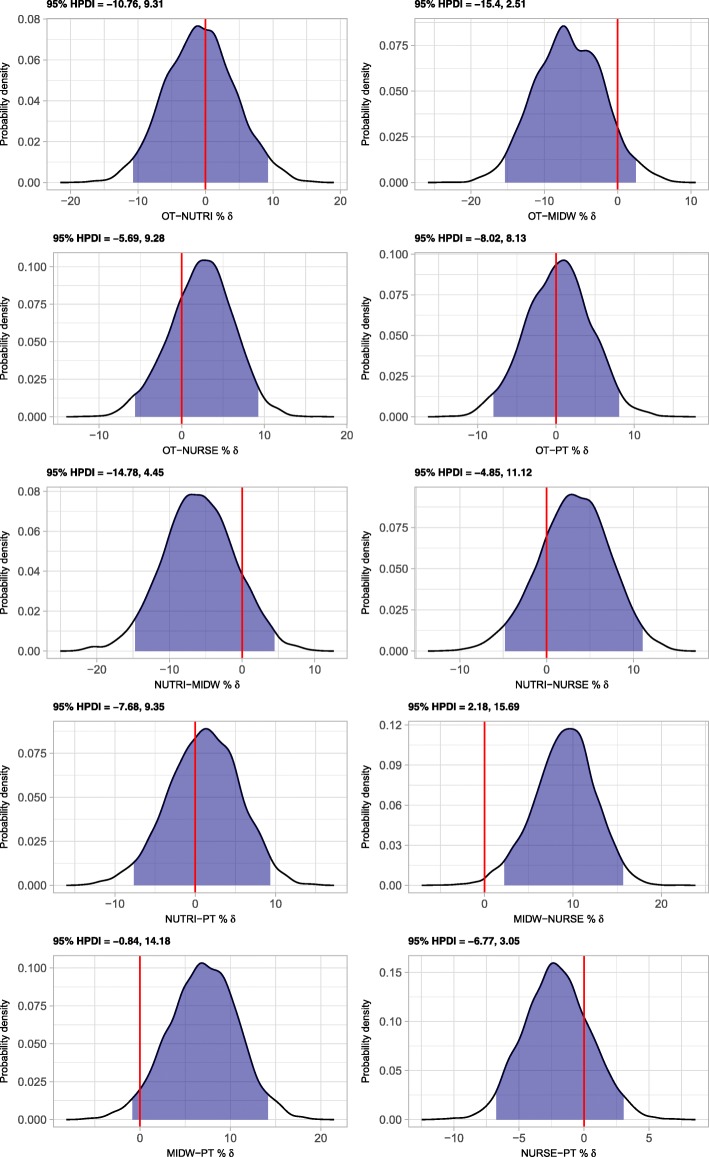
Probability density of differences in adjusted yearly prevalences of neck pain among health professions’ students. 95% HPDI: 95% highest posterior density interval. Source: Nat-ABBE coordination group, National Graduate Survey of Health Professionals from Universities of Applied Sciences 2016, 2017

The overall adjusted yearly prevalence of NP in HP students (Table 3) was 75.0% (72.8–77.1). Again, the prevalence was highest in students of midwifery (82.3% (76.1–88.1)). Students of the remaining professions all had very similar adjusted yearly prevalence of NP of roughly 75%. With respect to differences among HP students (Fig. 2), the probability of observing a lower adjusted yearly prevalence of NP in students of midwifery as compared to students of nursing or physiotherapy was 0.006 and 0.036, respectively.

**Table 3:** Yearly prevalence of neck pain in final year health professions students by profession

Profession	Crude prevalence (%)	95% HPDI	Adjusted prevalence (%)	95% HPDI
Occupational Therapy	75.3	69.7–80.5	75.6	69.8–81.0
Nutritional Sciences	76.2	69.2–83.1	76.4	68.6–83.4
Midwifery	83.4	78.7–87.6	82.3	76.1–88.1
Nursing	72.4	69.6–75.1	73.2	70.4–76.1
Physiotherapy	72.4	68.0–76.6	75.3	71.2–79.5
All	73.9	72.0–76.0	75.0	72.8–77.1

Adjusted prevalences: adjusted for age and gender. 95% HPDI: 95% highest posterior density interval

Source: Nat-ABBE coordination group, National Graduate Survey of Health Professionals from Universities of Applied Sciences 2016, 2017

## Discussion

We examined Swiss Health Survey data from 2012 and data from the National Survey of Final Year HP Students (2016 and 2017 cohorts combined) to estimate the prevalence of LBP and NP specific to Switzerland. Our results revealed worryingly high prevalence for both LBP and NP in final year HP students when compared to the general and demography-stratified Swiss population.

These results are particularly concerning for a group yet to embark on their careers in professions that may be deemed more physically hazardous than for many other professions. Beginning professional careers with disabling conditions like LBP and NP may threaten professional longevity and heighten the already projected workforce shortage. While the prevalence shown in the HP students studied are worrying enough, of additional concern is our finding that suggests that midwifery final year students are particularly susceptible to experiencing both conditions. We are unable to support causal relationships due to our study design, but contend that further research is urgently required to explain the high prevalence of LBP and NP in HP students compared to their national peers. Further, we have indicated inter-professional susceptibilities that necessitate further study in order that mitigating management strategies can potentially be introduced into curricula. Further, we suggest that students’ physical and mental health be recognized (and measured) as vital in developing competence for clinical practice.

The yearly prevalence for LBP (76%) and NP (75%) in final year HP students in our cohort is not only higher than the age, gender, and education-matched respondents to the SHS, but also apparently high when compared to mean values reported for professionals in clinical practice where yearly LBP is around 53% [26] and NP, 45% [27]. Further, our rates appear high compared to the literature for other HP students [20, 22, 23, 41–43], and particularly so for the midwifery students in our cohort who describe over 80% yearly prevalence for both conditions, which even exceeds accepted global lifetime prevalence estimates [3, 4, 9, 31]. While we remain cautious in comparing our values to global studies where various factors may differ (e.g. measures, timeframes and definitions), there can be no question our worrying findings warrant further study for explanation. In examining nursing and midwifery students, Williams & Crawford [44] report a 76% incidence of back pain, which we speculate may be elevated due to their inclusion of midwifery students who are shown, as one example, to attribute different meanings to varied pain descriptors [45]. In their study examining LBP prevalence in students from eight HPs (including medicine and dentistry but not midwifery), AlShayhan & Saadeddin [21] report their highest prevalence in dentistry students with 61%, which crude and adjusted prevalence for each of our professions exceeds (refer to Table 2). The present data-driven cross-sectional study was not designed to explain why this may be the case, but we acknowledge the rationale is multi-factorial and further exploration may best focus on the Swiss context given probable cultural and demographic influences. Furthermore, despite differing crude prevalence across studies, it will be important to understand whether similar patterns for professional susceptibilities exist in other countries [20–22], for example like in our nutritional sciences students where NP may represent more of a problem than LBP.

Not only are HPs affected themselves by the mounting burden of musculoskeletal disorders like LBP and NP they are collectively part of delivering treatment and prevention of such common health problems. While treating the public’s LBP and NP may lean more within one professional domain (e.g. physiotherapy) than another (e.g. nursing), it is sound professional practice for any clinician, irrespective of profession, to promote healthy behaviors in their patients/clients. For example, an occupational therapist working to improve a patient’s fine-motor hand skills will concurrently optimize the position and posture of the patient’s upper quadrant, including the head, neck and upper limb; further, they should be considering optimizing their own physical health in executing the intervention. However, certain professional practice environments (e.g. midwives tasked with delivering a baby within a client’s home) may not be easily controlled and therefore less conducive to healthy practice for the HP themselves. In this situation, the HP requires knowledge and awareness to assess the environment with their own health in mind, in addition to that of their patient [41].

Health promotion and prevention is an important part in education and training of the world’s HPs. In Switzerland, tertiary HP training programs are based on the CanMEDS Framework that defines seven different roles for HPs [46]. One of these roles is the “Health Advocate”, which is defined by the competency to “promote the health of individual patients, communities and populations” [46]. On this basis, we argue that training institutions should invest in inter-professional programs and/or taught curricula across their HPs that increase student and staff knowledge about such common and disabling conditions like LBP or NP. In particular, we contend that being able to identify their own or colleague behaviors that are known to predispose to LBP or NP, and how to avoid or compensate for them may be important in promoting long and enjoyable careers. Understanding HP students’ susceptibilities in relation to other professional student groups will be important to establish whether HP students are more attuned to musculoskeletal disorders, potentially based on their education. We consider a multi-professional exploration of student health status to be an important next step in extending our work.

Contrary to global wisdom, our findings indicate that 21–30 year old women with secondary or tertiary-education do not experience more LBP or NP than the general Swiss population (or at least respondents of the SHS). Rationale explaining this finding may be grounded in methodological limitations (described below), but may indicate a public health context unique to Switzerland; this strongly supports further study with targeted parameters relevant to Switzerland. Based on previous work undertaken by our group and others, young adults across Switzerland show wide variation in their health status [47] and appear at risk for declining self-reported health into the future [48]. It is clear from the findings of the present study that future research examining mechanisms that underpin LBP and NP in HP students in Switzerland is warranted, particularly in identifying inter-professional differences and risks that can be modified during the students’ formative professional education. As emerging clinicians with limited clinical exposure, HP students have relative inexperience in or awareness of the competencies needed to cope with physically demanding work practices, and may benefit from work-hardening measures that develop physical and manual handling attributes. As part of the wider competency framework study, students in our cohort will be followed at the first year after graduation, which allows for longitudinal examination of LBP and NP prevalence in the context of retention in the workforce. Prevalence of other health parameters surveyed in the NAT-ABBE will be examined in relation to healthcare usage/demand in further study.

While our study has strengths in what we believe to be the first to examine the prevalence of LBP and NP in HP students within a national context, employing a rigorous and contemporary statistical approach, and in revealing professional susceptibilities that warrant further qualitative and quantitative investigation, our findings should be considered in light of the study limitations.

Firstly, the SHS and NAT-ABBE survey were administered in 2012 and 2016/17 respectively. Consequently, period effects, while likely minimal, may bias our results because secular changes like lifestyle and teaching or studying practices between 2012 and 2016/17 could not be accounted for.

Secondly, while employing two Swiss datasets based on self-reported surveys with many similarities and that homogenize the context, the two surveys asked slightly different questions regarding LBP and NP and provided slightly different answer categories. In order to mitigate this problem, we derived dichotomous outcome variables that indicated the presence of absence of pain. However, collapsing response categories leads to a loss of information contained in the data. Moreover, simulation studies showed that collapsing outcome variables might affect the effect estimate as well as the inference being drawn from the data, particularly in a data set of limited size, i.e. less than 200 observations [33]. However, our sensitivity analyses found similarity of results regarding the size and statistical significance of effects yielded by a logistic model and an alternative cumulative odds model, which incorporated the original three (SHS) and four (Nat-ABBE) ordered categories of LBP and NP.

Thirdly, the SHS and NAT-ABBE survey referred to different time-frames, i.e. four-week and yearly prevalence of LBP and NP. In order to compare HP students with the general population, we used weights to convert yearly prevalence to four-week prevalence. While we admit that this is only a crude approximation of the “true” four-week prevalence, our sensitivity analysis showed that our results were still supported when weights were reduced by a substantial 25% percent.

Fourthly, several studies suggest that cultural and social factors affect the meaning of pain not only for patients but as well for health professionals [49–52]. Since HP students are socialized and educated within the cultural domain of the healthcare system, they may have adopted a different concept and understanding of pain compared to the general population. However, our study did not assess whether and to what degree different concepts of pain were associated with the prevalence of NP and LBP and future studies should address this issue.

## Conclusions

Swiss final year HP students when compared with the general and demography-stratified national population reported considerably higher LBP and NP. This worrying finding suggests high risk for LBP and NP in students of the clinical professions studied, and where midwifery may be particularly susceptible. Further study is urgently needed to explain these results and in order to institute mitigating strategies to improve student health outcomes. We contend that tertiary institutions are responsible for their students’ health in addition to developing student’s professional skills and competence in commencing clinical careers. Tertiary institutions should therefore provide education, awareness, and experiences that promote retention in the workforce, which we clearly show that musculoskeletal disorders may threaten. Whether health professions students represent a unique risk for these conditions should be examined against students studying other professions.

## Abbreviations

95% CI: 95% confidence intervals
95% HPDI: 95% highest posterior density interval
LBP: Low back pain
Nat-ABBE: National Graduate Survey of Health Professionals from Universities of Applied Sciences
NP: Neck pain
SFSO: Swiss Federal Statistical Office
SHS: Swiss Health Survey
